# Seasonal weather impacts wine quality in Bordeaux

**DOI:** 10.1016/j.isci.2023.107954

**Published:** 2023-10-11

**Authors:** Andrew Wood, Samuel J.L. Gascoigne, Gregory A. Gambetta, Elizabeth S. Jeffers, Tim Coulson

**Affiliations:** 1 Department of Biology, University of Oxford, 11a Mansfield Road, Oxford OX1 3SZ, UK; 2 EGFV, Bordeaux Sciences Agro, INRAE, Université de Bordeaux, ISVV, Villenave d’Ornon, France

**Keywords:** Climatology, Environmental science, Agricultural science, Data analysis

## Abstract

Critics judge quality based upon subjective characteristics of wine. These judgments are converted by critics into quantitative scores, which allow for comparison of vintages. This paper uses high resolution discrete and continuous time-based weather estimates at both a local and regional level to determine the role of weather conditions on producing high quality Bordeaux vintages, as determined by critics scores. By using discrete-time weather variables across local AOCs, this study reveals climate-quality relationships across the whole year, including previously ignored season effects. By using continuous time weather variables, we reinforce the evidence for these local effects by finding higher quality wine is made in years with higher rainfall, warmer temperatures; and earlier, shorter seasons. We propose management impacts of our results and suggest that as the climate continues to change, the quality of Bordeaux wines may continue to improve.

## Highlights

• Wine quality measured through critics scores varies across space and time
• Weather at multiple points during the year impacts wine quality in Bordeaux
• Climate shifts suggest wine quality may improve with future predicted climate

## Graphical abstract

**Figure:**
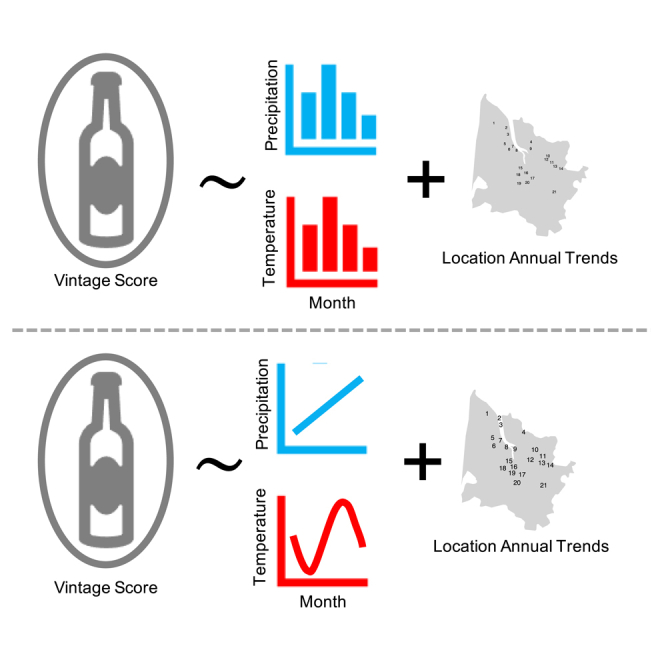

## Introduction

Climate change is globally impacting agricultural produce, both in terms of yield and quality.^1^^,^^2^ Despite these expected effects, the link between climate change and agricultural produce quality has not been widely explored. Wine (*Vitis vinifera*) presents the ideal system to study this relationship as wine price is governed primarily by quality,^3^ which is dependent on weather during the vine’s growing season.^4^ Additionally, wine quality in Bordeaux (France) has been measured by many independent experts over time, meaning that there exists a multi-critic regional and local longitudinal dataset for quality.^5^^,^^6^^,^^7^^,^^8^^,^^9^^,^^10^^,^^11^ With the availability of high-resolution weather data we can now use this information to examine how weather influences quality on both a regional and local scale.

Local variation in the quality of wine was first acknowledged with the introduction of wine rating systems. The Bordeaux Grand Cru system was created in 1855 to classify individual vineyards into one of five categories based on price and perceptions of quality. This Grand Cru classification system has been expanded such that there now exists 14 defined categories of wines in Bordeaux, with other wines simply being categorized as unclassified via this method.^11^ A series of geographical protections were introduced in 1936, referred to as *appellations d’origine contrôlée*, or AOCs. Acting on the local scale, they create individualities for wines, with each AOC having distinct viticultural characteristics and vinicultural identities.^12^ Such identities can link an AOC to perceived quality, with some becoming more famous than others. Regional and local disparities can be explored by comparing scores for the whole of Bordeaux to individual wine scores linked to an AOC. Consequently, each individual wine is wrapped in its own historic quality ratings which have the potential to shift perceptions of the current and future wines. Such perceptions must be considered in any attempt to understand quality.

Some studies have directly examined quality using tasting scores.^13^^,^^14^^,^^15^ In Bordeaux these tasting scores traditionally take the form of a *primeur* score. These scores are bestowed by wine critics at tastings approximately 10 months after harvest and just after blending. While these wines are not mature and often highly tannic,^11^ this scoring system provides a direct standardized measurement of quality and allows for an ascertainment of the quality of the wine before it fully ages. Other critics, mainly wine merchants, rate Bordeaux as a whole region, giving an overall classification as to whether or not a year is good or not. Due to this two-scale rating system, there exists the potential to compare regional tasting scores to local tasting scores.

Weather conditions have also been demonstrated to have an impact on the wine quality. Most famously, Ashenfelter’s (1995) Bordeaux equation^6^^,^^7^^,^^8^ suggested that the average price of wine in Bordeaux is a linear function of winter precipitation and summer temperature. Other models have used monthly weather, demonstrating that finer resolution weather data and local chateau characteristics^6^^,^^16^ can contribute to explaining price variation in Bordeaux wines. These local effects have been examined using this same price-based approach by Lecocq and Visser (2006) using local weather stations.^17^ The models in Lecoq and Visser (2006) found similar results at both regional and local scales and thus suggested that in most cases regional and local weather records are interchangeable.

Tasting scores have been correlated with single-year metrics of weather such as annual mean temperature and precipitation^7^^,^^9^^,^^18^ in wine-growing regions from Australia^14^ to California.^13^ Consensus from the Bordeaux equation, price modeling and current quality scores suggest that higher temperature and lower precipitation leads to higher quality grapes.^5^^,^^7^^,^^11^ Ashenfelter and Jones (2013) suggest that: critics scores “reflect qualitatively the same weather factors that have been documented to be determinants of wine quality.”^6^ Multiple studies have shown that a higher number of warm days during flowering and at the onset of berry ripening (spring and summer) and lower precipitation during berry maturation (autumn) leads to higher quality.^5^^,^^19^^,^^20^ But other conflicting studies have shown impacts outside of this time frame, with weather affecting quality across the year. Notably, the Bordeaux equation suggests that primarily winter precipitation and summer temperature is important. But Vittorio and Ginsburgh (1996) use the number of days of hail in April as well as temperature and precipitation during June to September.^16^ Jones and Storchman (2001) look at phenological stages and find that four different weather aspects (evapotranspiration; total rainfall; and the number of days with temperatures more than 25°C and 30°C) all have an effect on the price of the final wine.^11^ Baciocco et al. (2014) suggest that low rainfall and high heat accumulation over the year lead to higher ranked wines.^10^ Bonada et al. (2020) claim that rainfall during winter dormancy impacts quality.^21^ Alongside such varying insights in the literature comes a finding of a reduction in quality with high temperatures.^18^^,^^20^ Other evidence also links spring frosts to changes in quality.^22^ Overall, these findings suggests the potential for regional differences in climate change to potentially impact wine quality.^23^ Thus, it is important to understand which features of the weather are affecting wine quality and when, in order to determine the precise impacts of climate change on a viticulturally relevant spatial resolution.

In this study, we explore the link between weather and critic quality scores, using weather and quality scores for Bordeaux wines, at both regional and local levels. We use discrete time models with time steps such that the impact of temperature and precipitation on wine quality scores across the year can be ascertained. We then use continuous time models which explore the weather across the whole year as single functions for rainfall and temperature. In turn, we aim to give greater understanding as to when wine quality is most susceptible to changes in temperature and precipitation, and how we can examine such changes.

## Results

**Figure 1:**
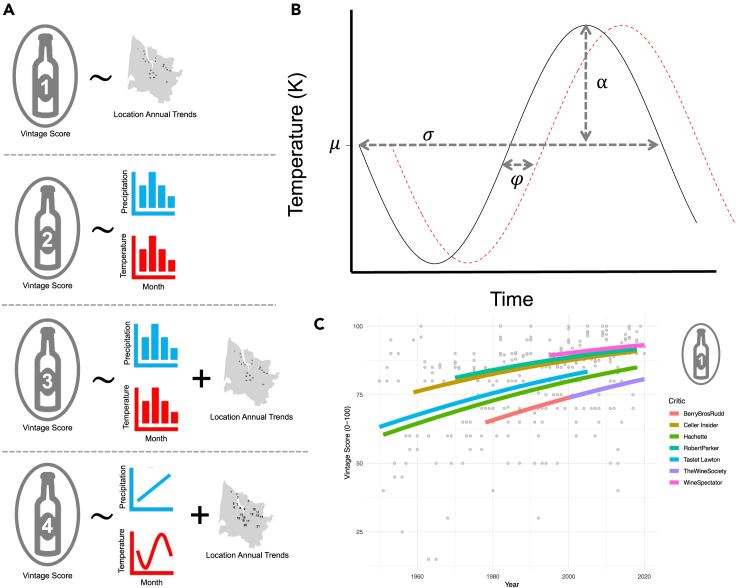
Methods panel plot (A) Summary of the generalized linear models run to analyze the relationship between wine critics scores and the weather, controlling for location annual trends (location and year interactions). In each row the number on the bottle refer to the model number, and tilde means “is a function of”. (B) Depiction of sine wave with depiction of parameters fitted. μm is the mean temperature, α the amplitude, σ the wavelength, and φ the phase shift. (C) Mean critics scores (scaled from 0 to 100) over time for Bordeaux as a whole with colored lines showing the GLM fitted.

Between 1950 and 2020 there was a general increase in quality scores for wine quality in the Bordeaux region. The maximal annual mean score was 98.67 points (1961), and the minimum was 32.5 (recorded in 1965). A generalized linear model was fitted to determine the location annual trends, as per model 1 in the model summary figure (Figure 1A). In this GLM, the overall critic score for Bordeaux is predicted by the year, controlling for critic. Year fitted as a continuous variable in the model was found to be statistically significant (coef = 0.0195, χ (1) = 4.4528, p < 0.05, R^2^ = 0.27), meaning there is a general increase in critics’ quality score over time (Table S3).

Quality scores were also examined on a local scale. The maximal mean critics score was 99 points (recorded once in 2019) and the minimum was 28 (recorded once in 2006). Critics showed high correlation between ratings (Figure S2). A binomial GLM was fitted, again as per model 1 on the model summary figure (Figure 1A). It reported no significant coefficients for Year nor Year:AOC interactions. This suggests that, for the time period, there is no local increase in quality over time (for test statistics see Table S4).

### Discrete time model

**Figure 2:**
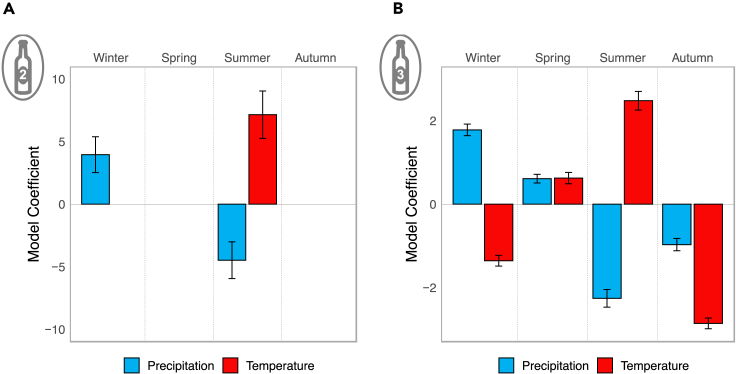
Weather variable model coefficients (A and B) Weather variable model coefficients for generalized linear models (GLMs) fitted to explain (A) mean critic score for the whole of Bordeaux and (B) mean critic score controlling for AOC, Grand Cru status, and year. Bar presence signifies coefficient had a p value of less than 0.05 with standard error bars.

Generalized linear models were also built to explore the relationship between grouped monthly weather variables and the mean overall Bordeaux general score, controlling for the yearly trend of improvements (R^2^ = 0.61; Table S5), as per model 2 in the model summary figure (Figure 1A). As weather has been normalized, only the sign (positive or negative) and the relative size of the coefficients are important. The largest significant (p < 0.05) coefficient in the model is the positive coefficient for summer temperatures (coef = −4.48, F (1,59) = 122.1716, p = 0.0004), followed by the negative coefficient for summer precipitation (coef = 7.14, F (1,59) = 114.0373, p < 0.05), and then the positive coefficient for winter precipitation (coef = 3.95, F (1,59) = 164.4203, df = 3, p < 0.05), as shown in Figure 2A. Models were found to fit well from visual inspection of residual plots (see Figure S3). According to this model, changes in temperature and precipitation at other times of year would not change the overall Bordeaux scores, excluding stochastic extreme weather events.

**Figure 3:**
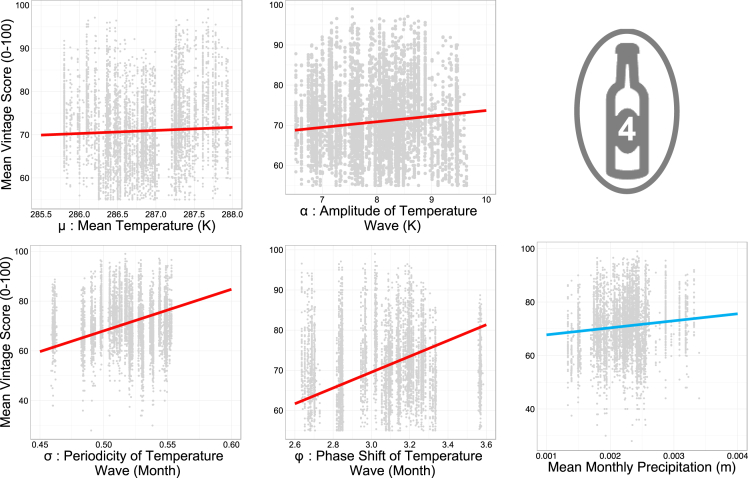
Model effects plots Model effects plot showing the vintage score against the sine wave parameters and mean monthly precipitation, with the slope of the line being the coefficients of each parameter.

A generalized linear model was also run on the local (AOC) scale, as per model 3 on the wine model summary figure (Figure 1A). Model 3 examines the relationship between grouped monthly weather variables and individual wine scores, controlling for year increases, AOC, and Grand Cru status (n = 4521, R^2^ = 0.35; for full details see Table S6). All weather terms were found to be significant, with coefficients shown graphically in Figure 3. Again, temperature and rainfall have been normalized for comparison purposes, and so exact coefficients are without real-world meaning. The largest coefficient is the negative term for temperature in autumn, followed by the positive coefficient for summer temperature, the positive term and the third largest impact is the negative coefficient for precipitation in summer. Models fitted well from visual inspection of plots (Figure S4). The variation in the coefficients shows the heterozygosity of the impacts, with positive and negative impacts occurring across the year. The coefficients appear to be in a wave formation, with both temperature and precipitation increasing and decreasing in a cyclical manner.

### Results for models using parameters from continuous time weather models

Cumulative precipitation increases linearly with increasing month (Figure S5). Over time there exists a generalized trend of increasing precipitation with increasing year (year coef = 0.0000318, F(1,6185) = 1676.8, p < 0.05, for full results see Table S7). When mean critic scores are modeled against cumulative precipitation controlling for AOC and year, as per model option 4 in Figure 1A, a positive correlation is found (coef = 1779.8, F(1,6171) = 28.1, n = 4521, p < 0.05, R^2^ = 0.29, Table S8). This suggests that higher whole year cumulative rainfall is beneficial for the production of higher quality wines.

Mean monthly temperatures have minima at around 3 to 4 months after September, in December and January. Peaks occur between 9- and 10-month past September—in June and July (Figure S6), increasing and decreasing in a wave pattern. Sine curves were fitted across each of the mean monthly temperatures and fitted the data well (mean R^2^ is 0.95 and standard deviation of R^2^ is 0.02, Table S9). μm and α were found to be the most variable terms, with means of 8.01 and 286.72 and standard errors of 0.79 and 0.65 (Table S9). σ and φ were found to have means of 0.52 and 3.07 and standard errors of 0.02 and 0.23 (Table S9). This suggests that mean temperature and temperature extremes can vary more than the timings of when seasons change.

Mean precipitation and the sine parameters of quality were used together as explanatory variables in a GLM, as per model 4 in Figure 1A. All factors, namely: mean precipitation (MeanPrecip), mean temperature (μ; C), amplitude (α; A), periodicity (σ, omega), and curve shift (φ; phi) were found to have significant (p < 0.05) positive coefficients (see Table S10 for test statistics). The marginal effects from this model can be seen plotted in Figure 3. These results suggest that the greater the extremes in temperature (higher amplitude), the shorter and earlier the season (decrease in wavelength and positive temperature phase shift), and the larger the mean temperature and mean monthly precipitation, the higher the mean critic score of the wine.

## Discussion

All of the models suggest that weather is an important factor in the determination of wine quality. Taken as a whole, the models suggest that weather impacts the quality of wine over the course of the year, with importance varying between seasons and aspects of seasonal weather. This paper includes a new method of examining the weather-wine linkage, using continuous time rather than discrete time periods. It concludes that different aspects of temperature and precipitation are important to quality throughout the year, with high quality requiring periods of both high and low temperature and precipitation. Exploring weather as a continuous series, we find that higher quality wine is made in years with greater temperature extremes; earlier, shorter seasons; and potentially a higher mean temperature.

At a regional scale, quality can be seen to have increased over the last several decades (Figure 1C). The cause of such a trend cannot be distinguished statistically on such a scale. Multiple factors all act in concert to improve wine critics scores, namely: climate change, increasing technology, increased positivity in critics reviews, and increased matching of wines consumer pref. ^4^^,^^28^^,^^29^. This increase in technology and consequential changes in wine characteristics since the 1960s have been described as the “Peynaudization” of Bordeaux wines.^30^ Controlling for such trends therefore allows for greater exploration of the impact of individual aspects of wine quality scores. At a local scale, no increase in trend can be seen to exist, and thus no universal increase in quality can be detected (Table S4). This may be due to the shorter time duration, variation in wine making techniques, or potentially even that the regional trends reflect critic regional sentiment rather than specific wines and thus no actualized trend exists.

The overall Bordeaux generalized linear model (model 2 in Figure 1A) suggests the traditional view of high winter precipitation, high summer temperatures and low precipitation in the summer and autumn lead to high quality grapes (Figure 2A). This combination of precipitation and sunshine has previously been termed the “Bordeaux Equation,”^7^^,^^8^ and has shaped the global understanding of grapevines.^29^^,^^31^ However, the weather location we used to examine these regional scores is in the city center of Bordeaux. This is an urban area not related to viticulture. This suggests that general regional weather has some effect, but that this is not the whole story.

Like the Bordeaux equation, our overall models also advocate for the impact of out of growing season effects too, corroborating statistically with findings from Bonada et al. (2020) that an increase water availability during the dormancy phase (in our case from precipitation and in their case due to irrigation) leads to an increase in quality.^21^ During winter the grapevines are experiencing dormancy, and a negative temperature coefficient here suggests a cooling period is required for high quality wine. The models also concur with previous findings that rainfall during the winter leads to higher quality,^17^^,^^21^ with agricultural suggestions that this may be due to lower soil salinity.^32^ It has been suggested that more rain in the winter could lead to a better water balance during the growing season, however, it has previously been shown that in Bordeaux only 3 of the last 70 years have started the growing season not at full water soil capacity.^2^ Flowering, fruit set, and potentially the onset of berry ripening (depending on the year) all occur in summer, with hotter and drier weather again being suggested to make high quality wine in this time period, potentially due to lower promotion of major grape diseases.^7^^,^^19^

The individual AOC model (model 3 in Figure 1A) suggests a more complex view of the relationship and quality. While the same relationships between weather and quality are there during the summer and autumn for temperature and precipitation, additional effects are also present (Figure 2B). These are the negative effects of temperature in winter and autumn and precipitation in autumn, as well as the positive effect in temperature and precipitation in spring. The higher number of significant time periods suggests that weather impacts occur right across the year, with impacts of weather on quality score potentially varying due to the phenological stage of the grapevine.^5^ As well as the dormancy effects, higher precipitation and higher temperatures in spring advocate for wetter and warmer weather for bud and leaf burst. Finally, cooler and drier weather is best for ripening in autumn and the optimal harvest to make a high-quality vintage. There is also an element of susceptibility suggested, with the impacts of water deficit on wine quality having greater impact on wine quality during the winter and summer months, and wine quality being more susceptible to temperatures in summer and autumn.

Combined, the two normalized weather models (models 2 and 3) suggest a difference between the regional and local levels. They suggest that heterogeneity at the local (AOC) level is being masked when only examining the regional level. They therefore suggest that, in order to improve the viticultural understanding and hence relevance of such modeling approaches, more local scale weather effects should be considered.

While these discrete-time suggestions are useful independently, they do not inform about time sequences of weather, which is exactly how it occurs. To explore this more fully, weather was treated as a continuous time variable. When examined in continuous time, monthly precipitation was found to be erratic and thus suited a cumulative approach, with increases being added and forming a linear accumulation. The fact that such an accumulation is well approximated by a linear model suggests an almost constant aseasonal pattern of precipitation, with the slope of this linear accumulation being the mean monthly precipitation. Conceptually, examining this mean monthly precipitation allows us to consider whether a whole year is wet or dry, rather than just the segments set out in the discrete GLMs. The significant precipitation term suggests that, even controlling for temperature, year, location, and class, a positive relationship exists between mean monthly precipitation and wine quality (Table S10). Wetter years appear to lead to higher quality wines. Coupled with the discrete time models (Figure 2), this model therefore suggests that this high rainfall should optimally occur post-harvest and pre-growth, during the dormancy period.

Temperature is not linear, rather it fluctuates according to seasonality and thus can be well approximated with a sine curve. Each of the parameters of the sine curve informs aspects of a temperature regime over the course of a year. In the GLM exploring the impact of temperature and precipitation parameters on mean quality score (again controlling for year, class, and AOC), the coefficients of each of the terms are found to be positive (Table S10). This suggests that, aside from being wetter, years that make higher quality wine are characterized by greater temperature extremes, with a higher mean temperature, and earlier, shorter seasons. While the increase in mean temperature concurs with previous research,^7^^,^^20^ more extreme weather suggests colder winters and hotter summers give higher quality. Earlier seasons suggests that consistently warmer weather during early phenological stages is also beneficial. Warmer weather means lower risk of frost, suggesting that damage to crops extends beyond losses and into quality.^22^^,^^33^ Warmer weather also potentially suggests that higher metabolic rates and higher photosynthetic rates lead to higher quality grapes. Shorter seasons suggest that the cooling of temperatures toward the end of the growing season may positively impact the ripening of grapes. With increases in both mean and extreme temperatures predicted across France,^34^ and changes in timing and length of growing season also predicted across growing regions,^35^ this leads to the potential suggestion that wine in Bordeaux may continue to improve over time.

Among these trends there still exists the question of the local versus regional disparities. The differences between the local and regional models in both their model coefficients and statistical significances suggest the impacts from the local scale are being masked when examined at the regional scale. This may be because of the individual differences in weather, or due to disparities in the wine making in each of the AOCs. For each of the models that are built, the Grand Cru classification system suggests significant differences between the classification levels, and similarly AOC level differences appear to exist (see Tables S6–S10). However, one potential source of variation in these data may be bias in expert opinion.^36^^,^^37^^,^^38^^,^^39^^,^^40^ Statistically, it is impossible with these data to fully disentangle wine bias from perceived quality. Future studies where the data for wine quality is both linked to local weather and also rated in a double-blind fashion, will be necessary to capture the degree to which expert bias informs or weakens our predictions.

We suggest that such variation in quality classification between regions has masked local variation in impacts of weather on quality across the year. While we accept that biases exist within wine, both for a specific locale or classification, we have also demonstrated a significant shared understanding of quality.

There are clear management implications of these findings, which result from trying to optimize the environment for vine growth and fruit composition. Our models suggest that water regime is critical in determining higher quality wines. Specifically, to increase wine quality, ideal conditions include high water abundance during the winter months and low water abundance in the summer, coupled with high temperatures. Climate change in Bordeaux will likely lead to more extreme weather, with variation depending on the location.^41^ While some places will be in drought, others will encounter less total rainfall punctuated with short heavy rain events.^41^ For red wine production we suggest that if irrigation were to be considered, it would be best to target the water regimes highlighted in this work: a well-replenished soil water profile over the winter months followed by moderate to severe water deficits during the summer months (depending on yield and wine style considerations). In cases where heavy rainfall could be an issue in summer, increased drainage, erosion control, or, at an extreme, rainfall covers could be necessary. With regard to temperature management, summer management strategies which promote localized higher temperatures are suggested (especially during the ripening period). This can be achieved by increasing defoliation around the berries to reduce shading, but caution is warranted to guard against exposing fruit to temperature extremes. Finally, we agree with common practices of avoiding frost damage by raising temperatures around the vines during the spring months. With predicted phenological and weather changes leading to hotter and earlier summers, our results suggest that average Bordeaux quality scores may continue to increase.

This paper has explored the impact of weather on wine, seeking to determine the optimal growing conditions for high quality Bordeaux vintages. It explores the infamous Bordeaux equation, finding that the equation works well for explaining regional patterns, but that for individual AOCs the weather impacts occur over the course of the year. Exploring this weather sequentially, this paper finds that higher quality wine is made in years with greater temperature extremes; earlier, shorter seasons; and a higher mean temperature. This all suggests that as climate change increases, the wine quality may continue to get better.

### Limitations of the study

We appreciate that the study was only conducted using ratings for Bordeaux chateaus, and that the corresponding limitations are therefore that we can only control for the winery at the winery level. We cannot control for the winemaker changing, or any potential changes in the exact plots used to make the wines. Finally, it is statistically impossible to tell the difference over time between improvements in wine due to climate and winemaker (and hence this trend has been removed from dataset). Despite these limitations, we have shown a robust trend within the dataset concerning the impact of seasonal weather on the quality of wines.

## STAR★Methods

### Key resources table

**Table:** 

REAGENT or RESOURCE	SOURCE	IDENTIFIER
**Deposited data**		

ERA-5 Land Hourly Weather Data	https://doi.org/10.24381/cds.e2161bac	
BordOverview Wine Database	https://www.bordoverview.com/	

**Software and algorithms**		

RStudio		
tidyverse packages		
Krigr package		

### Resource availability

#### Lead contact

Further information and requests for resources and reagents should be directed to and will be fulfilled by the lead contact, Andrew Wood (wood_and@hotmail.com).

#### Materials availability

This study did not generate new unique reagents.

### Method details

This study is an analysis of the linkage between two key weather variables: temperature and precipitation, and the critic scores at a regional and local (AOC) level for the Bordeaux region. As in previous approaches,^7^^,^^23^ analysis of the relationship between weather variables and wine quality scores are based upon the assumption that beneficial weather influences will lead to higher wine quality. The quality-weather interaction methodology can be split into two approaches. Both approaches fit critics scores against weather using a generalized linear model (GLM), but each uses different quantifications of weather as variables. The first approach uses the mean temperature and precipitation during discrete time-steps as the variables; the second approach uses the parameters of functions fitted to the temperature and precipitation data as the variables in statistical models of quality. All data extraction and analysis were undertaken in R version 4.2, using the *tidyverse*^24^ and *baseR* packages.

#### Weather data

Historical weather data were extracted from the ERA-5 land reanalysis weather dataset^25^ for each Bordeaux AOC region and central Bordeaux on a monthly time-step using the *Krigr* package^26^ (see Table S1 for AOCs and their locations). ERA-5 land is a high temporal and spatial resolution interpolated dataset which is available on a 0.1° grid at time scales varying from hourly to monthly since January 1950.^25^ Temperature and precipitation data were extracted on a monthly time-step for a 1km radius from the latitude and longitude point given per AOC for a period of January 1950 to December 2020. Temperature was measured in Kelvin (K), and precipitation in meters (m), both SI units for their respective measures. A growing season was defined as running from 1^st^ November to 31^st^ October, with harvest occurring at the end of the year. This aligns with standard growing season measurements (May-October) but extends them to include the overwintering effects (November-May).

Monthly weather variables were expectedly found to be strongly autocorrelated (Appendix 6), and thus unable to be used individually for building GLMs. Accordingly, months were grouped into autocorrelated groups, which could be roughly thought of as seasons. These groups were determined by those consecutive months by which the inter-month temperature or precipitation Pearson correlation coefficient was above 0.4. Winter was defined as being November and December, Spring as January to May, Summer as June to August, and Autumn as September and October.

A second way of dealing with this temporal autocorrelation is to describe temporal variation in the weather data to a continuous function. Precipitation is erratic, and hence to examine it in a continuous fashion, cumulative precipitation was used. The cumulative monthly precipitation was modeled linearly using a GLM, meaning that the cumulative precipitation can be approximated using the mean monthly precipitation.

Monthly mean temperature across the year were also described using a sine curve, as in Figure 1B. Non-linear least squares used to fit the data to the following equation:

αsin(σM+φ)+μ

Where M is the number of months since October, and α,σ,φ, and μ are parameters to be fitted. Figure 1B shows how each parameter relates to a part of the sine curve. Starting values for parameters were chosen based upon a fixed periodicity for σ and φ ($\sigma =\frac{2\pi }{12},\varphi =3)$. σ was chosen because of the annual cycle, hence division by twelve, and φ was chosen as October is 3 months after the approximate peak annual temperature. α and μ were fitted using the maximum, minimum and mean values of the temperature data per site and year using the equations: α=(max(t)−min(t))/2 ; μ=mean(t).

#### Quality data

Annual quality scores were collected on two scales, regional and local. Regional critic scores are based on the opinions of how Bordeaux performed as a whole, with individual variation largely ignored and general trends suggested.^20^ Local scores are based on individual wines, which are tasted *en primeur* and then rated based upon this premature wine.^27^ For each, publicly available wine critic, scores were transformed into a standardized 0–100 scale. Whole region scores were available for the period of 1950–2020 and were drawn from several sources,^9^^,^^20^ with additional data drawn from online vintage charts (see Table S2). Regional primeur critics scores for the period 2014–2020 were compiled by Bolomey Wijnimport^27^ and consist of published ratings from major wine experts from France, UK, US, the Netherlands and Germany. All publications were tested for correlation between their scores, and 14 wine publications were chosen based on having a Pearson Correlation Coefficient of more than 0.4 with at least 3 other selected publications. This cut-off was chosen as a liberal threshold for the inclusion of publications in our analysis. The chosen cut-off means that at least 16% of the variance in critics scores can be attributed to a shared understanding of quality across 3 other publications. These scores were standardized within publications such that each was on a 0 to 100 scale. If wines were rated by more than one publication (78% wines) mean standardized scores were taken.

### Quantification and statistical analysis

#### Generalized linear models

Generalised linear models (GLMs) were used to explore the relationship between weather and quality. A summary diagram of the models fitted can be found in Figure 1A. In the diagram, the number in the bottle refers to the model number, and tildes mean “is a function of”. GLMs were chosen because they are a flexible modeling approach which does not require any particular error structure or variance.

As per model 1 in the summary diagram (Figure 1A), mean quality scores over time were investigated using a GLM with a binomial distribution and logit link, controlling for the critic. This was due to the bounded nature of the scoring system (0–100) and decreasing variation in quality scores over time. This allows for a comparison of longer-term regional versus local trends over time. At a regional scale, the yearly trend interacted with region to determine the differential baseline trend over time.

For both the regional (model 2, Figure 1A) and local (model 3, Figure 1A) level scores, a GLM with a Gaussian distribution was fitted between the mean of the annual critic scores and the normalized temperature and precipitation during each weather grouping, controlling for year and, in model 3, the AOC and Grand Cru status of the vineyard. Normality of residuals was checked visually using a qqplot and residuals vs. fitted plot. The seasonal means of temperature and precipitation were normalized by subtracting the mean of the weather group and dividing by the standard deviation (Standard Score normalisation). This means that the GLM examined the proportional positive or negative impact of each of the variables on quality, rather than the absolute value. In normalizing such environmental data, comparisons could be made between the variables and therefore the relative contributions of each can be ascertained. The year was controlled for by adding it as a continuous variable, and, in the AOC model, the locality was also controlled for by adding it as a factor.

GLMs were also built to compare the mean vintage score with weather treated as a continuous function (model 4, Figure 1A). This model utilized an additive Gaussian GLM to examine how each of these variables explained the variance within the mean critic scores, controlling for AOC, year, and class. These models were run for both temperature and precipitation separately and then together. We also ran a GLM without year as an explanatory variable due to covariance.

## Data Availability

This paper analyses existing, publicly available data. All data is publicly available at locations referenced within the text. Wine data is available here: https://www.bordoverview.com/ and climate data from: https://cds.climate.copernicus.eu/cdsapp#!/dataset/10.24381/cds.68d2bb30?tab=overview as documented in the reference list and the key resources table. Any additional information required to reanalyse the data reported in this paper is available from the lead contact upon request.
