# Supplementary material for: Seasonal weather impacts wine quality in Bordeaux
Source: iScience. 2023 Oct 11;26(10):107954. doi: 10.1016/j.isci.2023.107954 (PMC10638477; doi:10.1016/j.isci.2023.107954)

iScience, Volume *26*

Supplemental information

Seasonal weather impacts wine quality in Bordeaux

Andrew Wood, Samuel J.L. Gascoigne, Gregory A. Gambetta, Elizabeth S. Jeffers, and Tim Coulson

Appendix For “Seasonal Climate Impacts Wine Quality in Bordeaux”

Supplementary Table 1: AOC locations used for weather data extraction, related to all Figures

Region	Latitude	Longditude
Bordeaux (City Centre)	44.841225	-0.5800364
Haut-Médoc	45.3745047	-0.8788471
Pomerol	44.9311234	-0.1999904
Moulis	45.0564852	-0.7736381
Listrac-Médoc	45.0736769	-0.7905774
Côtes de Bourg	45.0343476	-0.4954363
Côtes de Castillon	44.8706805	-0.0754746
Pauillac	45.1995626	-0.7484994
St-Estèphe	45.5925	0.6633333
Côtes de Bordeaux	44.7311086	-0.3187064
Graves	45.6500048	-0.1060768
Margaux	45.046232	-0.6728851
Pessac-Léognan	44.7294474	-0.5997267
Côtes de Blaye	45.127548	-0.4368037
Premières Côtes de Bordeaux	44.7790343	-0.461452
Médoc	44.5563705	-0.0385057
St-Emilion	44.8931452	-0.1560662
Canon-Fronsac	44.7007217	-0.5288963
St-Julien	44.817706	1.1745895
Lalande de Pomerol	44.9560756	-0.2104434
Fronsac	44.9229141	-0.2740925

Supplementary Table 2: Quality Critics, related to all Figures

Bordeaux Overall Quality Ratings	Bordeaux Individual Wine Quality Ratings
Berry Bros & Rudd	Wine Advocate (Robert Parker & Lisa Perotti Brown)
Hachette	Neal Martin
Tastet Lawton	Jancis Robinson
Wine Spectator	Tim Aitkin
Wine Society	TASTE (Michael Betanne & Thierry Desseauve)
	James Suckling
	Wine Cellar Insider (Jeff Leve)
	Decanter
	Revue de France
	Bordeaux Insider (Jane Anson)
	Le Point
	Perswijn
	Wien Wisser (Rene Gabriel)
	Wine Doctor (Chris Kissack)

Supplementary Figure 1: AOC scores over time, related to Figure 1

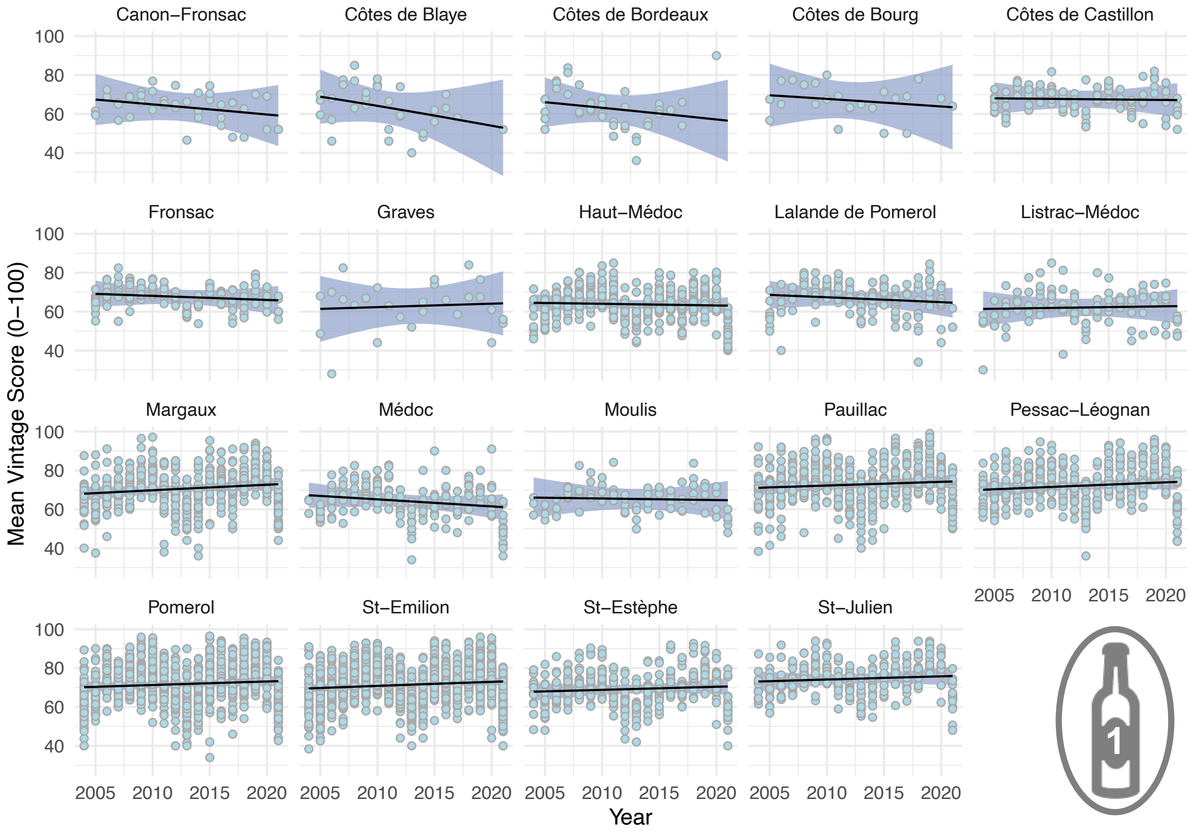

Supplementary Table 3: ANOVA table of GLM comparing quality increases over time across the whole region, related to Figure 1, panel C

glm(formula = MeanScore/100 ~ Year+Critic, family = binomial(link = "logit"))

Null deviance: 47.397 on 313 degrees of freedom
Residual deviance: 34.221 on 306 degrees of freedom
AIC: 204.98
R^2 = 0.2779923

ANOVA table:

term	df	χ-statistic	p.value	p-§
Year	1	4.4528	0.03484	Y
Critic	6	6.6488	0.35455	N

Supplementary Figure 2: Plot of individual critics scores plotted against each other and Pearsons correlations between them, related to Figure 1

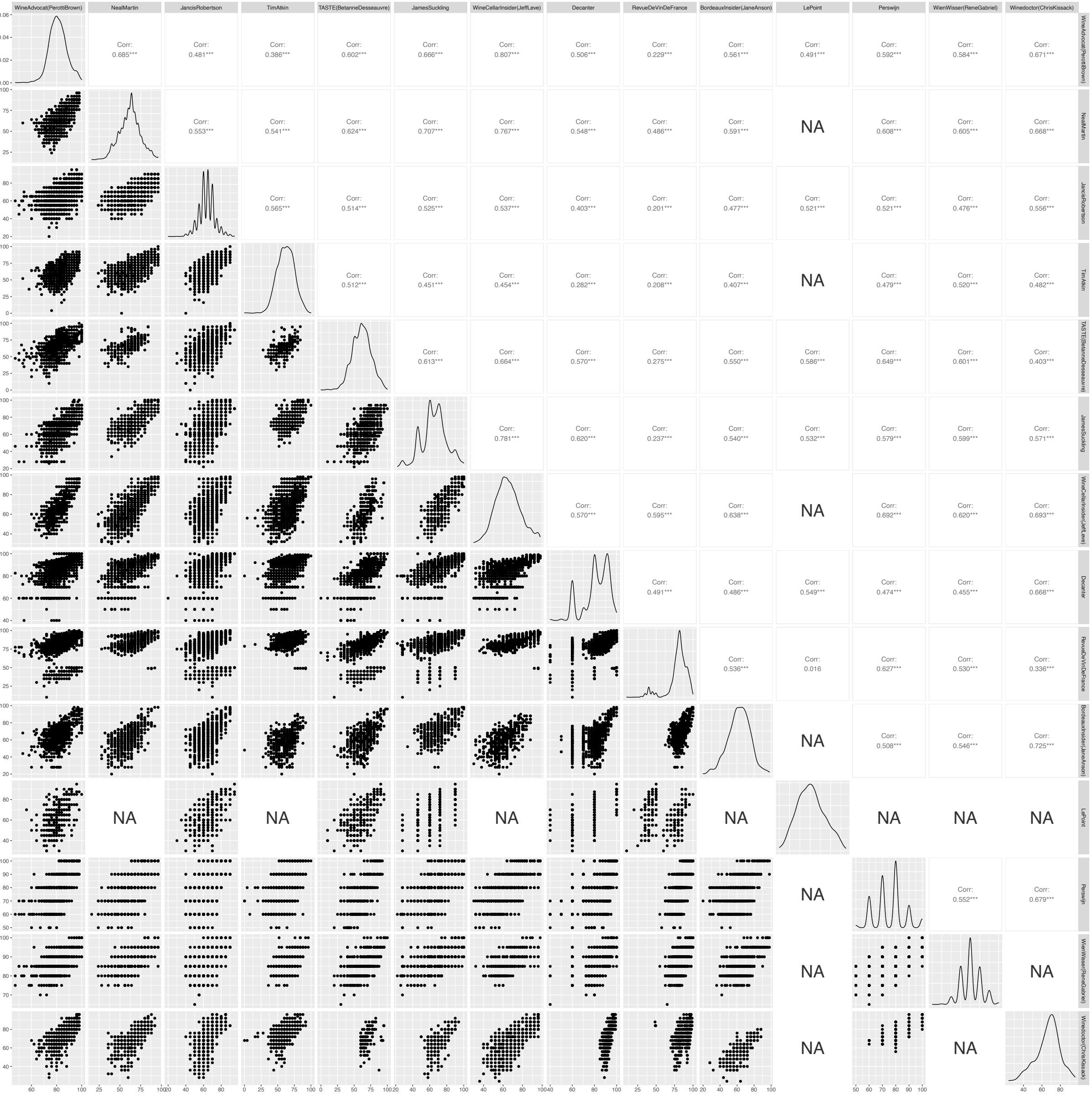

Supplementary Table 4: ANOVA Table of GLM comparing quality increases over time in different AOC regions, related to Figure 2

glm(formula = MeanVintageScore/100 ~ Year * AOC, family = binomial(link = "logit"))

Null deviance: 307.30 on 6566 degrees of freedom
Residual deviance: 273.44 on 6529 degrees of freedom
AIC: 5051
R^2 = 0.1101906

ANOVA table:

term	df	χ-statistic	p.value	p-value < 0.05
Year	1	1.3046	0.25337	N
AOC	18	30.2727	0.03488	Y
Year:AOC	18	2.5457	0.99999	N

Supplementary Table 5: ANOVA Table for Whole Bordeaux (Regional) Generalised Linear Model of Weather on Quality, related to Figure 2

glm(formula = MeanScore ~ Year + NormalisedWinPrecip + NormalisedSprPrecip + NormalisedSumPrecip + NormalisedAutPrecip + NormalisedWinTemp + NormalisedSprTemp + NormalisedSumTemp + NormalisedAutTemp, family = gaussian(), data = OverallGLMData, na.action = "na.omit")

Null deviance: 18587.0 on 68 degrees of freedom
Residual deviance: 7190.9 on 59 degrees of freedom
AIC: 538.42
R^2 = 0.6131224

ANOVA table:

term	sumsq	df	F-statistic	p.value	p-value < 0.05
Year	14.2161105	1	0.11664064	0.73392035	N
NormalisedWinPrecip	926.652503	1	7.60301804	0.00774375	Y
NormalisedSprPrecip	56.7666213	1	0.46575997	0.49761512	N
NormalisedSumPrecip	1133.65482	1	9.30143505	0.00342646	Y
NormalisedAutPrecip	214.658287	1	1.76123285	0.18958385	N
NormalisedWinTemp	3.74231222	1	0.030705	0.86150013	N
NormalisedSprTemp	286.087625	1	2.34729779	0.13084528	N
NormalisedSumTemp	1738.03832	1	14.2602936	0.00037225	Y
NormalisedAutTemp	259.55029	1	2.12956371	0.14978582	N
Residuals	7190.89411	59			

Supplementary Figure 3: Goodness of fit plots for overall generalised linear model for weather against quality score controlling for year, related to Figure 2

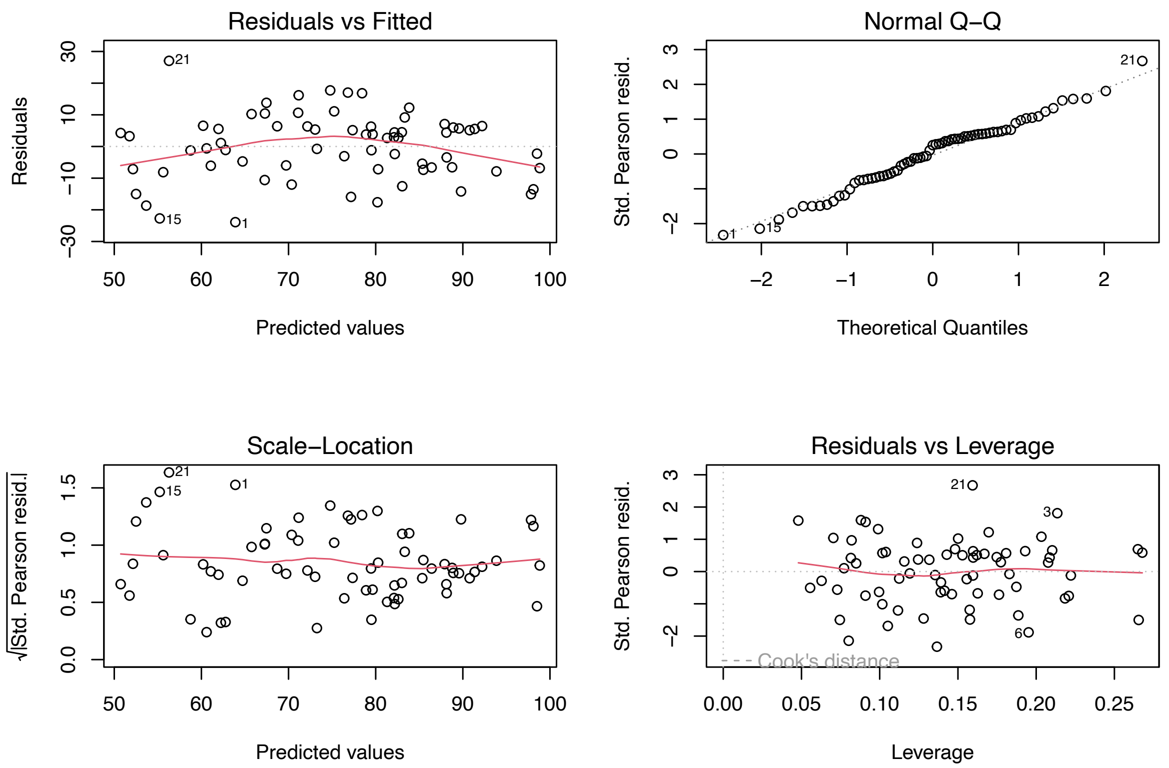

Supplementary Table 6: ANOVA Table for AOC (Local) Scores Generalised Linear Model of Weather on Quality, related to Figure 2

```
glm(formula = MeanVintageScore ~ AOC * Year + Class + NormalisedWinPrecip +
   NormalisedSprPrecip + NormalisedSumPrecip + NormalisedAutPrecip +
   NormalisedWinTemp + NormalisedSprTemp + NormalisedSumTemp +
   NormalisedAutTemp, family = gaussian(),
   na.action = "na.omit")
```

Null deviance: 560005 on 6222 degrees of freedom
Residual deviance: 362802 on 6163 degrees of freedom
AIC: 43082
R^2 = 0.3521448

ANOVA Table:

term	sumsq	df	F-statistic	p.value	p-value < 0.05
AOC	22855.9498	18	21.5699504	5.62E-69	Y
Year	2050.56773	1	34.8334506	3.78E-09	Y
Class	74847.8129	14	90.8183186	2.96E-238	Y
NormalisedWinPrecip	9679.06002	1	164.420347	3.64E-37	Y
NormalisedSprPrecip	2019.30686	1	34.3024151	4.96E-09	Y
NormalisedSumPrecip	6713.12406	1	114.037333	2.17E-26	Y
NormalisedAutPrecip	2523.45657	1	42.8665185	6.33E-11	Y
NormalisedWinTemp	6515.22182	1	110.675523	1.15E-25	Y
NormalisedSprTemp	1248.50272	1	21.2085936	4.20E-06	Y
NormalisedSumTemp	7191.97238	1	122.171636	3.89E-28	Y
NormalisedAutTemp	29009.5685	1	492.791998	4.23E-105	Y
AOC:Year	6149.22928	18	5.80324037	4.59E-14	Y
Residuals	362802.098	6163			

Supplementary Figure 4: Goodness of fit plots for local generalised linear model for weather against quality score controlling for year, AOC, and class, related to Figure 2

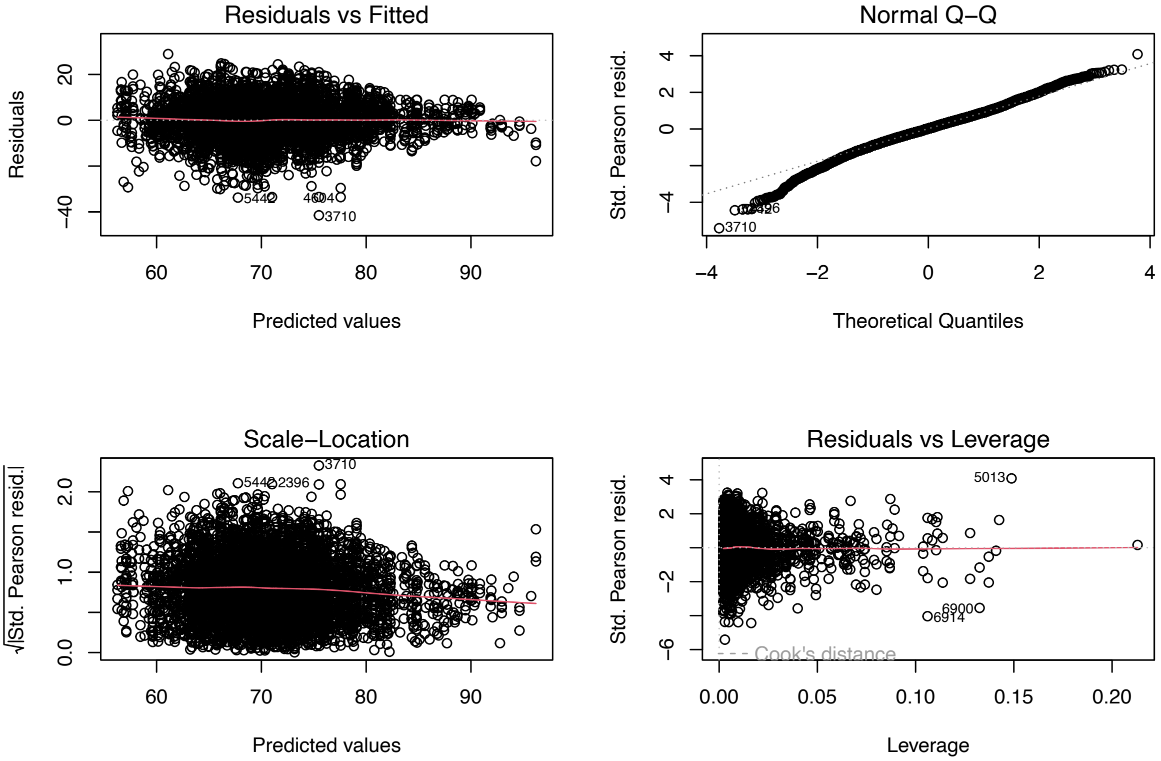

Supplementary Figure 5: Cumulative Monthly Precipitation Across all AOC of Bordeaux (1950-2020), related to Figure 3

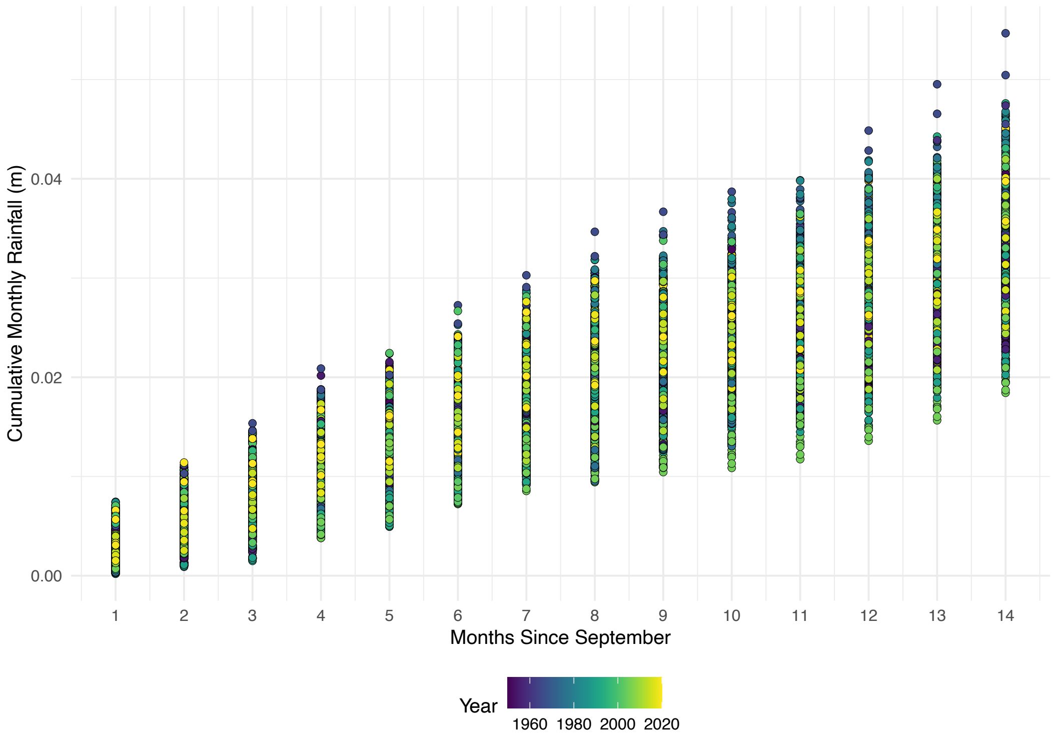

Supplementary Figure 6: Mean Monthly temperature across all AOCs in Bordeaux (1950-2020), related to Figure 3

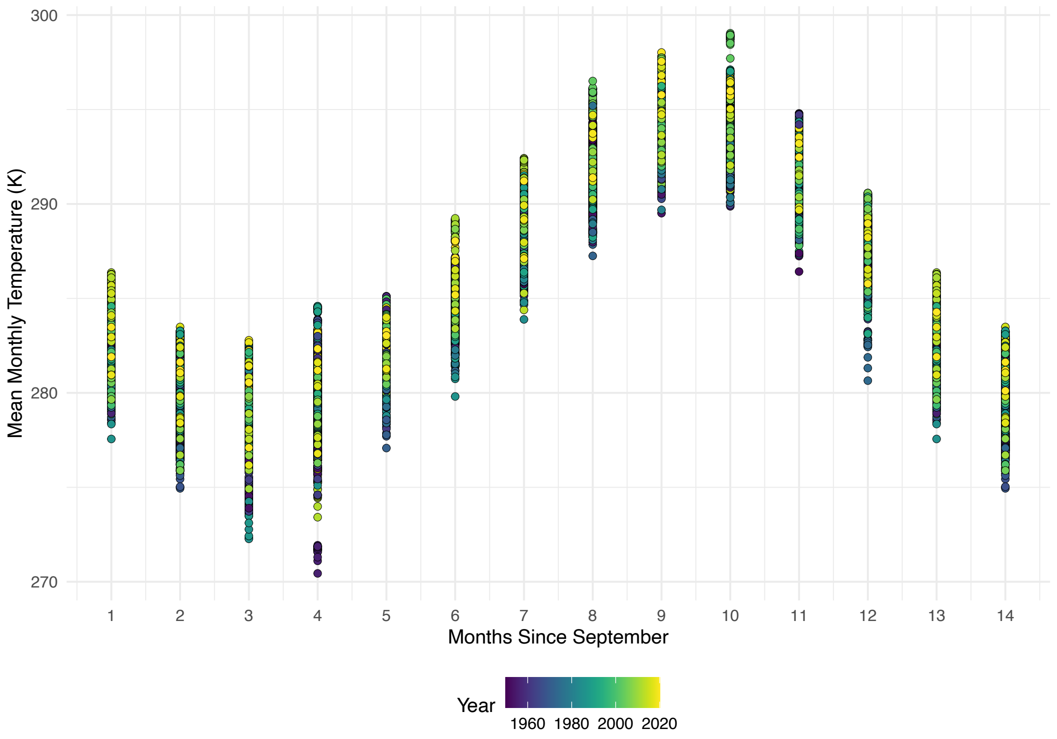

Supplementary Table 7: ANOVA Table For Precipitation-Year GLM, related to Figure 3

glm(formula = MeanPrecip ~ AOC * Year)

Null deviance: 0.00063460 on 4819 degrees of freedom
Residual deviance: 0.00048849 on 4784 degrees of freedom
AIC: -63872
R^2 = 0.2302302

ANOVA table:

term	sumsq	df	f-statistic	p.value	p-value < 0.05
AOC	5.93E-06	18	3.39182943	1.50E-06	Y
Year	0.00016285	1	1676.79052	0	Y
AOC:Year	3.97E-06	18	2.26854534	0.00164684	Y
Residuals	0.00060068	6185			

Supplementary Table 8: ANOVA Table for Generalised Linear Model of Vintage Score and Precipitation, related to Figure 3

glm(formula = MeanVintageScore ~ MeanPrecip + AOC * Year)

Null deviance: 421986 on 4520 degrees of freedom
Residual deviance: 347495 on 4484 degrees of freedom
AIC: 32536
R^2 = 0.1765253

ANOVA table:

term	sumsq	df	f-statistic	p.value	significance
MeanPrecip	1813.6452	1	28.0821828	1.20E-07	Y
Class	74404.4808	14	82.290478	7.97E-217	Y
AOC	24729.4977	18	21.2726366	6.25E-68	Y
Year	8669.74509	1	134.240901	1.00E-30	Y
AOC:Year	6801.80703	18	5.85100314	3.21E-14	Y
Residuals	398480.097	6170	NA	NA	

Supplementary Table 9: Mean and standard deviation of sine curves fitted to monthly temperature data (1950-2020), related to Figure 3

$$\alpha \sin(\sigma M + \varphi) + \mu$$

α (A)		σ (omega)		φ (phi)		μ (C)		R^2	
Mean	SD	Mean	SD	Mean	SD	Mean	SD	Mean	SD
8.01	0.79	0.52	0.024	3.07	0.23	286.7156	0.65	0.954	0.017

α >> amplitude
σ >> periodicity, length of waves
φ >> phase shift (positive = earlier)
μ >> mean temperature

Supplementary Table 10: Model ANOVA Table for GLM of mean vintage score described by mean precipitation and sine parameters of temperatures, related to Figure 3

```
glm(formula = MeanVintageScore ~ A + AOC + C + Class + MeanPrecip +
   omega + phi + Year + AOC:Year + 1, family = gaussian())
```

Null deviance: 425248 on 4520 degrees of freedom
Residual deviance: 291112 on 4469 degrees of freedom
AIC: 31766
R^2 = 0.3154307

ANOVA table:

term	sumsq	df	F-statistic	p.value	p-value significance
A	4149.6994	1	66.4808914	4.24E-16	Y
AOC	24050.1539	18	21.4055086	2.13E-68	Y
C	256.8952	1	4.11562868	0.04253162	Y
Class	75250.522	14	86.1116037	1.82E-226	Y
MeanPrecip	3328.49824	1	53.3247131	3.18E-13	Y
omega	9461.38421	1	151.577547	1.98E-34	Y
phi	12153.4513	1	194.706219	1.37E-43	Y
Year	3536.46822	1	56.6565279	5.93E-14	Y
AOC:Year	6253.52076	18	5.56586011	2.72E-13	Y
Residuals	384878.21	6166	NA	NA	

<u>Supplementary Figure 7: Goodness of Fit For Model, related to Figure 3</u>

GLM predictions plotted (orange, with error in purple) against data points (black). Vertical dotted lines indicate the mean predicted values. Plotted to test goodness of fit

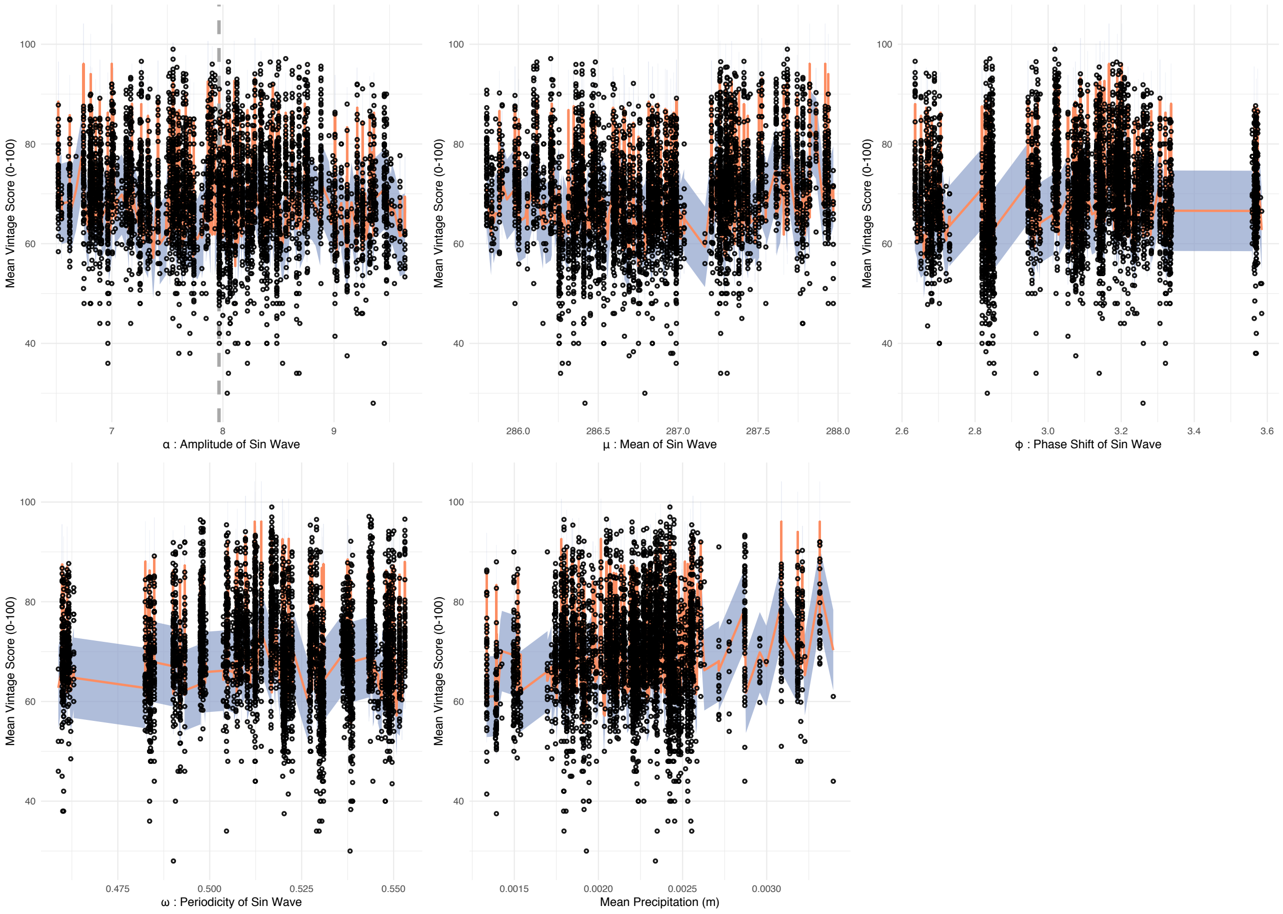

Supplement: Document S1. Figures S1–S7 and Tables S1–S10 [file mmc1.pdf]
